# Clustering COVID-19 ARDS patients through the first days of ICU admission. An analysis of the CIBERESUCICOVID Cohort

**DOI:** 10.1186/s13054-024-04876-5

**Published:** 2024-03-21

**Authors:** Adrian Ceccato, Carles Forne, Lieuwe D. Bos, Marta Camprubí-Rimblas, Aina Areny-Balagueró, Elena Campaña-Duel, Sara Quero, Emili Diaz, Oriol Roca, David De Gonzalo-Calvo, Laia Fernández-Barat, Anna Motos, Ricard Ferrer, Jordi Riera, Jose A. Lorente, Oscar Peñuelas, Rosario Menendez, Rosario Amaya-Villar, José M. Añón, Ana Balan-Mariño, Carme Barberà, José Barberán, Aaron Blandino-Ortiz, Maria Victoria Boado, Elena Bustamante-Munguira, Jesús Caballero, Cristina Carbajales, Nieves Carbonell, Mercedes Catalán-González, Nieves Franco, Cristóbal Galbán, Víctor D. Gumucio-Sanguino, Maria del Carmen de la Torre, Ángel Estella, Elena Gallego, José Luis García-Garmendia, José Garnacho-Montero, José M. Gómez, Arturo Huerta, Ruth Noemí Jorge-García, Ana Loza-Vázquez, Judith Marin-Corral, Amalia Martínez de la Gándara, María Cruz Martin-Delgado, Ignacio Martínez-Varela, Juan Lopez Messa, Guillermo Muñiz-Albaiceta, María Teresa Nieto, Mariana Andrea Novo, Yhivian Peñasco, Juan Carlos Pozo-Laderas, Felipe Pérez-García, Pilar Ricart, Ferran Roche-Campo, Alejandro Rodríguez, Victor Sagredo, Angel Sánchez-Miralles, Susana Sancho-Chinesta, Lorenzo Socias, Jordi Solé-Violan, Fernando Suarez-Sipmann, Luis Tamayo-Lomas, José Trenado, Alejandro Úbeda, Luis Jorge Valdivia, Pablo Vidal, Jesus Bermejo, Jesica Gonzalez, Ferran Barbe, Carolyn S. Calfee, Antonio Artigas, Antoni Torres, CIBERESUCICOVID Project, Berta Adell-Serrano, María Aguilar Cabello, Luciano Aguilera, Victoria Alcaraz-Serrano, Cesar Aldecoa, Cynthia Alegre, Raquel Almansa, Sergio Álvarez, Antonio Álvarez Ruiz, Rosario Amaya Villar, Ruth Andrea, Mariana Andrea Novo, José Ángel, Jose Manuel Añon, Marta Arrieta, JIgnacio Ayestarán, Joan Ramon Badia, Mariona Badía, Orville Báez Pravia, Ana Balan Mariño, Begoña Balsera, Carme Barberà, José Barberán, Laura Barbena, Enric Barbeta, Tommaso Bardi, Patricia Barral Segade, Marta Barroso, José Ángel Berezo García, Jesús F. Bermejo-Martin, Belén Beteré, Judit Bigas, Aaron Blandino Ortiz, Rafael Blancas, María Luisa Blasco Cortés, María Boado, María Bodi Saera, Neus Bofill, María Teresa Bouza Vieiro, Leticia Bueno, Elena Bustamante-Munguira, Juan Bustamante-Munguira, Cecilia del Busto Martínez, Jesús Caballero, David Campi Hermoso, Sandra Campos Fernández, Cristina Carbajales, Iosune Cano, Maria Luisa Cantón-Bulnes, Nieves Carbonell, Pablo Cardina Fernández, Laura Carrión García, Sulamita Carvalho, Núria Casacuberta-Barberà, Manuel Castellà, Andrea Castellví, Pedro Castro, Mercedes Catalán-González, Ramon Cicuendez Ávila, Catia Cillóniz, Luisa Clar, Cristina Climent, Jordi Codina, Pamela Conde, Sofía Contreras, María Cruz Martin, Raul de Pablo Sánchez, Diego De Mendoza, Emili Díaz, Yolanda Díaz, María Digna Rivas Vilas, Cristina Dólera Moreno, Irene Dot, Pedro Enríquez Giraudo, Inés Esmorís Arijón, Angel Estella, Teresa Farre Monjo, Javier Fernández, Carlos Ferrando, Albert Figueras, Eva Forcadell-Ferreres, Lorena Forcelledo Espina, Nieves Franco, Enric Franquesa, Àngels Furro, Albert Gabarrus, Cristóbal Galbán, Elena Gallego, Felipe García, Beatriz García, José Luis García Garmendia, Dario Garcia-Gasulla, Emilio García Prieto, Carlos García Redruello, Amaia García Sagastume, José Garnacho-Montero, Maria Luisa Gascón Castillo, Gemma Gomà, José M. Gómez, Vanesa Gómez Casal, Silvia Gómez, Carmen Gómez Gonzalez, David de Gonzalo-Calvo, Jessica González, Federico Gordo, Maria Pilar Gracia, Víctor D. Gumucio-Sanguino, Alba Herraiz, Rubén Herrán-Monge, Arturo Huerta, Mercedes Ibarz, Silvia Iglesias, Maria Teresa Janer, Gabriel Jiménez, Ruth Noemí Jorge García, Mar Juan Díaz, Karsa Kiarostami, Juan ILazo Álvarez, Miguel León, Alexandre López-Gavín, Ana López Lago, Juan Lopez Messa, Esther López-Ramos, Ana Loza-Vázquez, Desire Macias Guerrero, Nuria Mamolar Herrera, Rafael Mañez Mendiluce, Cecilia L. Mantellini, Gregorio Marco Naya, Pilar Marcos, Judith Marin-Corral, Enrique Marmol Peis, Paula Martín Vicente, María Martínez, Carmen Eulalia Martínez Fernández, Amalia Martínez de la Gándara, Maria Dolores Martínez Juan, Basilisa Martínez Palacios, Ignacio Martínez Varela, Juan Fernando Masa Jimenez, Joan Ramon Masclans, Emilio Maseda, Eva María Menor Fernández, Mar Miralbés, Josman Monclou, Juan Carlos Montejo-González, Neus Montserrat, María Mora Aznar, Dulce Morales, Sara Guadalupe Moreno Cano, David Mosquera Rodríguez, Rosana Muñoz-Bermúdez, Guillermo Muñiz Albaiceta, José María Nicolás, Maria Teresa NIeto, Ramon Nogue Bou, Rafaela Nogueras Salinas, Marta Ocón, Ana Ortega, Sergio Ossa, Pablo Pagliarani, Francisco Parrilla, Jose Pedregosa-Díaz, Yhivian Peñasco, Oscar Peñuelas, Leire Pérez Bastida, Purificación Pérez, Felipe Pérez-García, Gloria Pérez Planelles, Eva Pérez Rubio, David Pestaña Laguna, Àngels Piñol-Tena, Javier Prados, Andrés Pujol, Juan Carlos Pozo, Núria Ramon Coll, Gloria Renedo Sanchez-Giron, Jordi Riera, Pilar Ricart, Ferran Roche-Campo, Alejandro Rodríguez, Laura Rodriguez, Felipe Rodríguez de Castro, Silvia Rodríguez, Covadonga Rodríguez Ruiz, Jorge Rubio, Alberto Rubio López, Ángela Leonor Ruiz-García, Miriam Ruiz Miralles, Pablo Ryan Murúa, Eva Saborido Paz, Victor Sagredo, Ana Salazar Degracia, Inmaculada Salvador-Adell, Miguel Sanchez, Ana Sánchez, Angel Sánchez-Miralles, Susana Sancho Chinesta, Bitor Santacoloma, Miguel Sanchez, Maria Teresa Sariñena, Marta Segura Pensado, Lidia Serra, Mireia Serra-Fortuny, Ainhoa Serrano Lázaro, Lluís Servià, Lorenzo Socias, Laura Soliva, Jordi Solé-Violan, Fernando Suarez Sipmann, Carla Speziale, Luis Tamayo Lomas, Adrián Tormos, Maria del Carmen de la Torre, Gerard Torres, Mateu Torres, Sandra Trefler, Josep Trenado, Javier Trujillano, Alejandro Úbeda, Luis Urrelo-Cerrón, Estela Val, Manuel Valledor, Luis Valdivia Ruiz, Montserrat Vallverdú, Maria Van der Hofstadt Martin-Montalvo, Sabela Vara Adrio, Nil Vázquez, Javier Vengoechea, Pablo Vidal, Clara Vilà-Vilardel, Judit Vilanova, Tatiana Villada Warrington, Hua Yang, Minlan Yang, Ana Zapatero

**Affiliations:** 1 Critical Care Center, Hospital Universitari Parc Taulí, Institut d’Investigació i Innovació Parc Taulí (I3PT-CERCA), Department of Medicine, Universitat Autonoma de Barcelona, Plaça Torre de L’Aigua, S/N, 08208 Sabadell, Spain; 2 Centro de Investigación Biomédica en Red en Enfermedades Respiratorias (CIBERES), Instituto de Salud Carlos III, Madrid, Spain; 3 Intensive Care Unit, Hospital Universitari Sagrat Cor, Grupo Quironsalud, Barcelona, Spain; 4 Heorfy Consulting, Lleida, Spain; 5 Department of Basic Medical Sciences, University of Lleida, Lleida, Spain; 6 Intensive Care and Laboratory of Experimental Intensive Care and Anesthesiology (LEICA), Amsterdam UMC Location AMC, University of Amsterdam, Meibergdreef 9, Amsterdam, The Netherlands; 7 Translational Research in Respiratory Medicine, Respiratory Department, Hospital Universitari Aranu de Vilanova and Santa Maria, IRBLleida, Lleida, Spain; 8 Department of Pneumology, Hospital Clinic of Barcelona, August Pi i Sunyer Biomedical Research Institute–IDIBAPS, University of Barcelona, Barcelona, Spain; 9 Intensive Care Department, Hospital Universitari Vall d’Hebron, Vall d’Hebron Institut de Recerca, Barcelona, Spain; 10 Hospital Universitario de Getafe, Universidad Europea, Madrid, Spain; 11 Department of Bioengineering, Universidad Carlos III, Madrid, Spain; 12 Pulmonary Department, University and Polytechnic Hospital La Fe, Valencia, Spain; 13 Intensive Care Clinical Unit, Hospital Universitario Virgen de Rocío, Seville, Spain; 14 Servicio de Medicina Intensiva, Hospital Universitario La Paz, IdiPAZ, Madrid, Spain; 15 Hospital Universitario San Agustín, Asturias, Spain; 16 Hospital Santa Maria, IRBLleida, Lleida, Spain; 17 Hospital Universitario HM Montepríncipe, Facultad HM Hospitales de Ciencias de La Salud, Universidad Camilo Jose Cela, Madrid, Spain; 18 Servicio de Medicina Intensiva, Hospital Universitario Ramón y Cajal, Madrid, Spain; 19 Intensive Care Unit, and Emergency Medicine, Universidad de Alcalá, Madrid, Spain; 20 Hospital Universitario de Cruces, Barakaldo, Spain; 21 Department of Intensive Care Medicine, Hospital Clínico Universitario Valladolid, Valladolid, Spain; 22 Critical Intensive Medicine Department, Hospital Universitari Arnau de Vilanova de Lleida, IRBLleida, Lleida, Spain; 23 Intensive Care Unit, Hospital Álvaro Cunqueiro, Vigo, Spain; 24 Intensive Care Unit, Hospital Clínico Universitario, Valencia, Spain; 25 Department of Intensive Care Medicine, Hospital Universitario, 12 de Octubre, Madrid, Spain; 26 Hospital Universitario de Móstoles, Madrid, Spain; 27 Department of Critical Care Medicine, CHUS, Complejo Hospitalario Universitario de Santiago, Santiago, Spain; 28 Department of Intensive Care, Hospital Universitari de Bellvitge, Bellvitge Biomedical Research Institute (IDIBELL), L’Hospitalet de Llobregat, Barcelona, Spain; 29 Hospital de Mataró de Barcelona, Barcelona, Spain; 30 Department of Medicine, Intensive Care Unit University Hospital of Jerez, University of Cádiz, INIBiCA, Cádiz, Spain; 31 Unidad de Cuidados Intensivos, Hospital Universitario San Pedro de Alcántara, Cáceres, Spain; 32 Intensive Care Unit, Hospital San Juan de Dios del Aljarafe, Seville, Spain; 33 Intensive Care Clinical Unit, Hospital Universitario Virgen Macarena, Seville, Spain; 34 Hospital General Universitario Gregorio Marañón, Madrid, Spain; 35 Pulmonary and Critical Care Division, Emergency Department, Clínica Sagrada Família, Barcelona, Spain; 36 Intensive Care Department, Hospital Nuestra Señora de Gracia, Saragossa, Spain; 37 Unidad de Medicina Intensiva, Hospital Universitario Virgen de Valme, Seville, Spain; 38 Critical Care Department, Hospital del Mar-IMIM, Barcelona, Spain; 39 Department of Intensive Medicine, Hospital Universitario Infanta Leonor, Madrid, Spain; 40 Hospital Universitario Torrejón-Universidad Francisco de Vitoria, Madrid, Spain; 41 Critical Care Department, Hospital Universitario Lucus Augusti, Lugo, Spain; 42 Complejo Asistencial Universitario de Palencia, Palencia, Spain; 43 Departamento de Biología Funcional, Instituto Universitario de Oncología del Principado de Asturias, Instituto de Investigación Sanitaria del Principado de Asturias, Hospital Central de Asturias, Universidad de Oviedo, Oviedo, Spain; 44 Hospital General de Segovia, Segovia, Spain; 45 Servei de Medicina Intensiva, Hospital Universitari Son Espases, Palma, Illes Balears Spain; 46 Servicio de Medicina Intensiva, Hospital Universitario Marqués de Valdecilla, Santander, Spain; 47 UGC-Medicina Intensiva, Hospital Universitario Reina Sofia, Instituto Maimonides IMIBIC, Córdoba, Spain; 48 Servicio de Microbiología Clínica, Facultad de Medicina, Departamento de Biomedicina y Biotecnología, Hospital Universitario Príncipe de Asturias - Universidad de Alcalá, Alcalá de Henares, Madrid, Spain; 49 Centro de Investigación Biomédica en Red en Enfermedades Infecciosas (CIBERINFEC), Instituto de Salud Carlos III, Madrid, Spain; 50 Servei de Medicina Intensiva, Hospital Universitari Germans Trias, Badalona, Spain; 51 Institut d’Investigació Sanitària Pere Virgili (IISPV), Hospital Verge de la Cinta, Tortosa, Tarragona, Spain; 52 Critical Care Department, Hospital Universitario Joan XXIII, CIBERES, Rovira and Virgili University, IISPV, Tarragona, Spain; 53 Hospital Universitario de Salamanca, Salamanca, Spain; 54 Intensive Care Unit, Hospital Universitario Sant Joan d’Alacant, Sant Joan d’Alacant, Alicante, Spain; 55 Servicio de Medicina Intensiva, Hospital Universitario y Politécnico La Fe, Valencia, Spain; 56 Intensive Care Unit, Hospital Son Llàtzer, Illes Balears, Palma, Spain; 57 Critical Care Department, Hospital Universitario de GC Dr. Negrín, Universidad Fernando Pessoa Canarias, Las Palmas, Gran Canaria Spain; 58 Intensive Care Unit, Hospital Universitario La Princesa, Madrid, Spain; 59 Critical Care Department, Hospital Universitario Río Hortega de Valladolid, Valladolid, Spain; 60 Servicio de Medicina Intensiva, Hospital Universitario Mútua de Terrassa, Terrassa, Barcelona, Spain; 61 Servicio de Medicina Intensiva, Hospital Punta de Europa, Algeciras, Spain; 62 Hospital Universitario de León, León, Spain; 63 Complexo Hospitalario Universitario de Ourense, Orense, Spain; 64 Hospital Universitario Río Hortega de Valladolid, Valladolid, Spain; 65 Instituto de Investigación Biomédica de Salamanca (IBSAL), Gerencia Regional de Salud de Castilla y León, Salamanca, Spain; 66 Division of Pulmonary, Critical Care, Allergy and Sleep Medicine, Department of Medicine, University of California, San Francisco, San Francisco, CA USA

**Keywords:** ARDS, Clustering, Mortality, Precision medicine

## Abstract

**Background:**

Acute respiratory distress syndrome (ARDS) can be classified into sub-phenotypes according to different inflammatory/clinical status. Prognostic enrichment was achieved by grouping patients into hypoinflammatory or hyperinflammatory sub-phenotypes, even though the time of analysis may change the classification according to treatment response or disease evolution. We aimed to evaluate when patients can be clustered in more than 1 group, and how they may change the clustering of patients using data of baseline or day 3, and the prognosis of patients according to their evolution by changing or not the cluster.

**Methods:**

Multicenter, observational prospective, and retrospective study of patients admitted due to ARDS related to COVID-19 infection in Spain. Patients were grouped according to a clustering mixed-type data algorithm (k-prototypes) using continuous and categorical readily available variables at baseline and day 3.

**Results:**

Of 6205 patients, 3743 (60%) were included in the study. According to silhouette analysis, patients were grouped in two clusters. At baseline, 1402 (37%) patients were included in cluster 1 and 2341(63%) in cluster 2. On day 3, 1557(42%) patients were included in cluster 1 and 2086 (57%) in cluster 2. The patients included in cluster 2 were older and more frequently hypertensive and had a higher prevalence of shock, organ dysfunction, inflammatory biomarkers, and worst respiratory indexes at both time points. The 90-day mortality was higher in cluster 2 at both clustering processes (43.8% [*n* = 1025] versus 27.3% [*n* = 383] at baseline, and 49% [*n* = 1023] versus 20.6% [*n* = 321] on day 3). Four hundred and fifty-eight (33%) patients clustered in the first group were clustered in the second group on day 3. In contrast, 638 (27%) patients clustered in the second group were clustered in the first group on day 3.

**Conclusions:**

During the first days, patients can be clustered into two groups and the process of clustering patients may change as they continue to evolve. This means that despite a vast majority of patients remaining in the same cluster, a minority reaching 33% of patients analyzed may be re-categorized into different clusters based on their progress. Such changes can significantly impact their prognosis.

**Supplementary Information:**

The online version contains supplementary material available at 10.1186/s13054-024-04876-5.

## Background

Acute respiratory distress syndrome (ARDS) is a heterogeneous condition characterized by respiratory failure and diffuse pulmonary noncardiogenic edema [1]. ARDS is associated with poor short- and long-term outcomes including high mortality rates, disability, and poor quality of life indicators. Several trials have failed to identify treatments for ARDS, and only corticosteroids [2], prone position [3], and limiting the damage caused by mechanical ventilation [4, 5] have shown improved outcomes.

The COVID-19 pandemic hit Europe in late winter/early spring 2020, probably resulting in the highest number of ARDS patients ever treated concomitantly. COVID-19 has increased the knowledge about the physiopathology and treatment of ARDS, highlighting the importance of anti-inflammatory treatment over antivirals [6–9], thromboembolic events [10], and endothelial damage [11].

Sub-phenotypes of ARDS have been described as hypoinflammatory and hyperinflammatory, using clinical data and plasma inflammatory biomarkers in latent class analysis [12]. These sub-phenotypes have been associated with different outcomes, and reanalyses of several clinical trials found that the hyperinflammatory sub-phenotype may have better outcomes when receiving simvastatin [13], or high-positive end-expiratory pressure (PEEP) levels [12]. In addition, these sub-phenotypes responded differently to fluid management [14]. Models for a parsimonious identification of sub-phenotypes using a few variables and machine-learning strategies are being developed. However, during the COVID-19 pandemic, fewer patients could be classified into the hyperinflammatory sub-phenotype [15]. A possible limitation of clustering is that the correct identification of sub-phenotypes is needed, and only one cross-sectional evaluation may underestimate the impact of the changes or evolutions of markers in the first days of admission. Bos et al. failed to identify sub-phenotypes in patients with COVID-19 by cross-sectional analysis with data from the respiratory system but two sub-phenotypes were identified through longitudinal analysis [16]. Few data evaluating clustering using inflammation data and common variables measuring organ failure are available [17, 18].

We hypothesized that patients with COVID-19-associated ARDS may be described by more than one discrete cluster and the clustering process and its result may change during the first days of their admission, which may have an impact on their prognosis. To evaluate this, we aimed to study a cohort of patients and analyze the clustering of patients at baseline and day 3 using readily available data measuring organ failure, respiratory variables, and inflammation.

## Methods

### Study design

CIBERESUCICOVID is a multicenter, observational, prospective, retrospective cohort study that enrolled patients with COVID-19 admitted to Spanish intensive care units (ICUs) [19]. The study was approved by the institution’s Internal Review Board (Comité Ètic d’Investigació Clínica, registry number HCB/2020/0370), and written informed consent was obtained from either the patients or their relatives. Local researchers were contacted by a member of the study team, and the participating hospitals obtained approval from the local ethics committee. De-identified patient data were collected and stored via the REDCap electronic data capture tool [20, 21] hosted at the Centro de Investigación Biomédica en Red (CIBER), Spain. Trained local researchers incorporated data from the patient’s medical records into a separate database. Before statistical analyses, the data were checked by three independent experienced data collectors trained in critical care, and site investigators were contacted for any queries. Missing analyses were performed, and site investigators were contacted to obtain as much reliable and complete data as possible. Results were reported following the Strengthening the Reporting of Observational Studies in Epidemiology (STROBE) guidelines [22].

### Study population and data collection

All consecutive patients admitted to the ICU at a participating center from February 25, 2020, to September 30, 2021, were enrolled if they fulfilled the following criteria: ≥ 18 years of age, admission to the ICU, and laboratory-confirmed SARS-CoV-2 infection. For this study, we selected those patients with a diagnosis of ARDS based on the Berlin definition [23] who needed invasive mechanical ventilation during the first day of admission to the ICU and who remained ventilated at least 3 days later. Patients were excluded if they had a non-confirmed SARS-CoV-2 infection, had no data at baseline, or were admitted to the ICU for other reasons.

After enrollment, prior epidemiological data, including demographics, comorbidities, clinical symptoms, disease chronology, and treatment administered upon hospital admission, were collected. The site researchers subsequently collected data acquired at hospital admission, ICU admission, start of mechanical ventilation (MV), 72–96 h after ICU admission, weaning, ICU discharge, and hospital discharge, including vital signs, respiratory support devices (i.e., oxygen mask, high-flow nasal cannula, and noninvasive and invasive mechanical ventilation), use of adjunctive therapies (i.e., neuromuscular blockade, prone position, and recruitment maneuvers), laboratory findings, arterial blood gases, and mechanical ventilation settings, if appropriate. Hemodynamic parameters and organ dysfunction were assessed using the Sequential Organ Assessment Failure Score (SOFA) [24] at ICU admission. Pharmacological treatments administered upon and during ICU admission until discharge from the ICU or hospital or death were also collected.

Specific data regarding MV since the start of invasive mechanical ventilation and on day three were analyzed. MV parameters related to ventilation-induced lung injury (VILI), such as tidal volume, respiratory rate, end-inspiratory plateau, peak inspiratory pressures, positive end-expiratory pressure (PEEP), driving pressure, and static compliance of the respiratory system (Crs) were collected. Oxygenation impairment was analyzed using the PaO2/FiO2 ratio, and abnormalities in CO2 metabolism were studied using the ventilatory ratio (VR), a surrogate parameter of Vd/Vt. The worst event values were recorded preferentially. We did not analyze data from inflammatory cytokines.

### Definitions

The diagnosis of ARDS was based on the Berlin definition [23]. Tidal volume was reported in mL/kg of predicted body weight (PBW). The driving pressure was defined as plateau pressure minus PEEP. Crs was calculated as tidal volume/plateau pressure—PEEP. The ventilatory ratio was defined as follows: (minute ventilation × PaCO2)/(PBW × 100 × 37.5).

### Outcome

The primary outcome was the 90-day mortality.

### Statistical analysis

To derive the clusters, we first evaluated the distributions, missingness, and correlation of the candidate variables (Additional file 1: Fig. S1). Multiple imputation with chained equations was used to account for missing data, the results from the first dataset are presented, and the additional datasets were evaluated for consistency. After evaluating correlation, we excluded highly correlated variables (|rho|> 0.5) and those variables with more than 60% of missing values. Ordering Points To Identify the Clustering Structure plots were used to determine the optimal clustering strategy [25]. Based on these plots, we applied the k-prototypes clustering to 44 variables (age, gender, height, weight, chronic heart failure, diabetes mellitus, obesity, hypertension, hematologic disease, cancer, immunosuppression, systolic blood pressure, mean blood pressure, shock, temperature, heart rate, respiratory rate, pH, Hco3, hemoglobin, white blood counts, lymphocytes, platelet, D-dimer, prothrombin time, C-reactive protein, bilirubin, lactate, LDH, sodium, potassium, creatinine, procalcitonin, albumin, ventilatory ratio, tidal volume/ predicted body weight, driving pressure, Pao2/FiO2, PEEP, peak pressure, neuromuscular blocking agents use, prone position, ECMO) collected at baseline and replicated with data collected at day 3 (33 of 44 variables changed their values according to variable evolution) using a partitioning approach. The optimal number of clusters was determined based on the Silhouette index [26, 27]. Once the optimal number of clusters was determined, patterns of clinical variables were visualized in two ways: (1) chord plots (showing how clusters differ based on categorical variables) and (2) standardized values of each continuous variable by cluster.

To understand the implications of clusters and changes of clusters on 90-day mortality, Kaplan–Meier survival curves and hazard ratios adjusted by SOFA score were estimated, with 95% confidence intervals (CI).

In order to assess whether the clusters were explained by well-established measures of disease severity, we fitted logistic regression models including the SOFA score and/or the most influential variables in clustering as independent variables, and the C-indexes were obtained.

Data were presented as means and standard deviations (SD) or medians and percentiles of 25% and 75%. For comparisons between clusters, we used t tests and the Mann–Whitney U test for continuous data and the *χ*^2^ test for categorical data. The statistical significance was set at 0.05 for two-sided tests (without adjustment for multiple comparisons; therefore, statistically significant differences between clusters should be considered exploratory). Analysis was performed with the R statistical package version 4.3.1 (R Foundation for Statistical Computing).

## Results

**Fig. 1:**
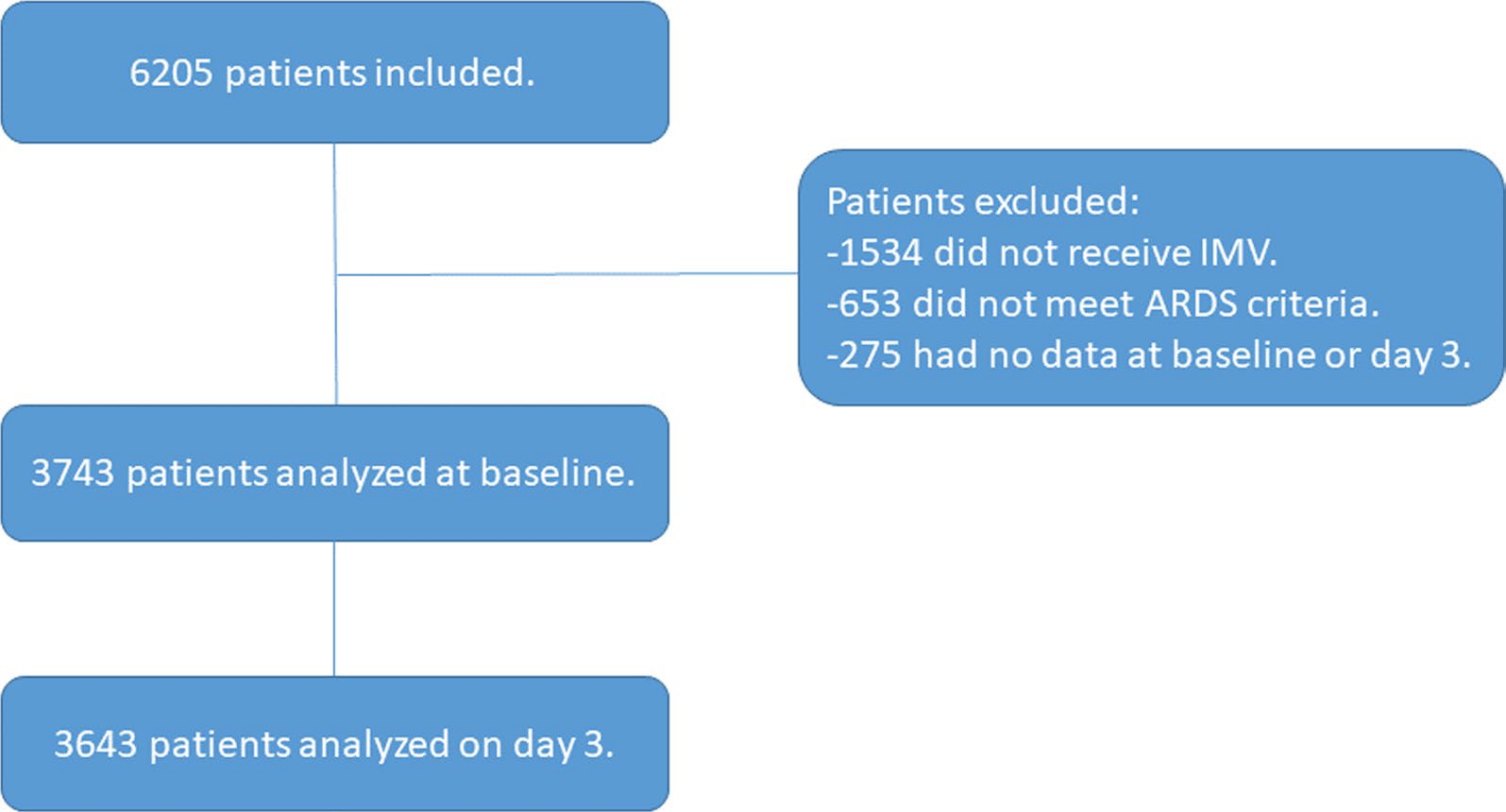
Flowchart of patient screening and enrollment

**Fig. 2:**
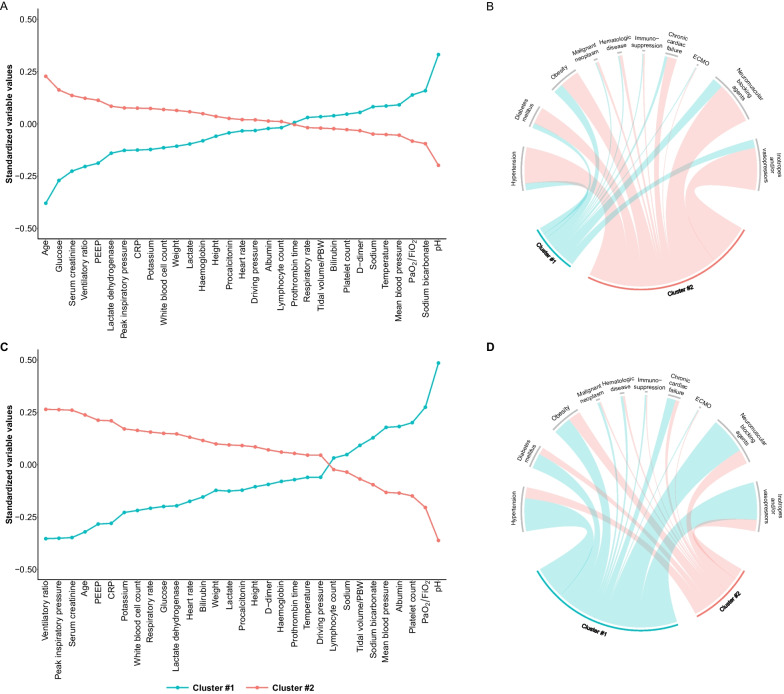
Comparison of variables that contribute to clusters. **A** Standardized values of each continuous variable by cluster at baseline. **B** Chord plots (showing how clusters differ based on categorical variables) at baseline. **C** Standardized values of each continuous variable by cluster at day 3. **D** Chord plots (showing how clusters differ based on categorical variables) at day 3. *CRP* C-reactive protein; *FiO*_*2*_ fraction of inspired oxygen; *IL* Interleukin; *MV* mechanical ventilation; *NT-proBNP* N-terminal pro-brain natriuretic peptide; *PaCO*_*2*_ arterial partial pressure of carbon dioxide; PaO_2_, partial pressure of arterial oxygen; PBW, predicted body weight; SOFA: sequential organ failure assessment score. ^a^Defined as plateau pressure—PEEP. ^b^Defined as tidal volume/ (Plateau pressure − PEEP). ^c^Defined as (minute ventilation × PaCO_2_)/ (PBW × 100 × 37.5)

Out of 6205 eligible patients, 3743 (60%) patients were included in the study (Fig. 1). Following the Silhouette statistic (Additional file 1: Fig. S3), patients were grouped into two clusters using the k-prototypes algorithm. At baseline, 1402 (37%) patients were included in cluster 1 and 2341 (63%) in cluster 2. The baseline characteristics of the patients are presented in Table 1. The patients included in cluster 2 were older, had more hypertension, shock, inflammation measured by CRP, and better respiratory indexes (including the requirement of the prone position, NMBA requirement, oxygenation by Pao2/Fio2, ventilatory ratio, and driving pressure) (Table 1). On day 3, 3643 patients were analyzed, 1557 (42%) patients were included in cluster 1 and 2086 (57%) in cluster 2. One hundred patients (3%) died or were discharged before day 3. The weight of each continuous variable in the clusters is shown in Fig. 2 as standardized variable values and counts for categorical variables. In the clustering process between baseline and day 3, several variables changed their influence with an evolution of the values for each variable, but at both moments the clinical characteristics of the clustering were similar.

**Table 1:** Demographic and clinical characteristics of patients by clusters at baseline and day 3

	Missing values, *n* (%) at baseline/*n* (%) at day 3	Clustering at baseline		Clustering on day 3	
		Cluster 1	Cluster 2	Cluster 1	Cluster 2
Age, years	4 (0.11%)	60 [50,69]	67 [60,72]	61 [51,69]	67 [60,73]
Sex, female	4 (0.11%)	494 (35%)	614 (26%)	574 (37%)	505 (24%)
Active smoker	730 (19.5)	68 (6.09%)	134 (7.06%)	81 (6.40%)	115 (6.93%)
Hypertension	15 (0.40%)	306 (22%)	1633 (70%)	463 (30%)	1420 (68%)
Diabetes mellitus	19 (0.51%)	221 (16%)	761 (33%)	296 (19%)	659 (32%)
Obesity	14 (0.37%)	459 (33%)	890 (38%)	529 (34%)	790 (38%)
Chronic heart failure	359 (9.59%)	112 (9%)	380 (18%)	145 (10%)	327 (18%)
Chronic kidney disease	11 (0.29%)	49 (3%)	171 (7%)	50 (3%)	157 (7%)
Chronic respiratory disease	11 (0.29%)	92 (7%)	280 (12%)	113 (7%)	246 (12%)
Hematologic disease	402 (10.7%)	48 (4%)	108 (5%)	66 (5%)	85 (5%)
Malignant neoplasm	394 (10.5%)	40 (3%)	78 (4%)	44 (3%)	72 (4%)
Immunosuppression	411 (10.9%)	24 (2%)	42 (2%)	20 (2%)	42 (2%)
APACHE II score at ICU admission	1743 (46.6%)	12 [9, 16]	14 [10, 19]	–	–
SOFA score	1411 (37.7%)/1685 (45%)	5 [4, 7]	7 [6, 8]	4 [3, 6]	7 [6, 8]
Temperature, ºC	649 (17.3%)/875 (23.4%)	36.9 [36.1,37.7]	36.8 [36.0,37.6]	36.7 [36.0,37.2]	36.8 [36.0,37.5]
Respiratory rate, bpm	765 (20.4%)/1011 (27%)	24 [20, 31]	25 [20, 30]	20 [18, 24]	23 [20, 26]
Systolic blood pressure, mmHg	589 (15.7%)/925 (24.7%)	122 [105,140]	120 [101,140]	130 [113,151]	125 [109,146]
Mean blood pressure, mmHg	1376 (36.8%)/1510 (40.3%)	85 [72,98]	83 [70,96]	90 [75,103]	82 [71,96]
Heart rate, bpm	620 (16.6%)/876 (23.4%)	85 [70,100]	87 [70,102]	70 [55,89]	80 [60,98]
PaO_2_/FIO_2_ ratio, mmHg	310 (8.28%)/359 (9.59%)	119 [83,177]	110 [80.6,154]	198 [156,250]	161 [122,210]
PaO2 mmhg	204 (5.45%)/328 (8.76%)	84 [67.0,114]	84[66.9,110]	88 [75,110]	86 [73,107]
Vasopressor use	419 (11.2%)/555 (14.8%)	352 (28%)	1718 (82%)	444 (32%)	1434 (79%)
pH	107 (2.86%)/310 (8.28%)	7.40 [7.34,7.45]	7.34 [7.27,7.41]	7.43 [7.40,7.46]	7.37 [7.31,7.42]
PaCO_2_, mmHg	117 (3.13%)/305 (8.15%)	41 [35.0,47.9]	45 [37.1,53.8]	43 [39.0,47.3]	48 [42.6,55.4]
Lactate, mmol/L	817 (21.8%)/1001 (26.7%)	1.40 [1.10,1.90]	1.50 [1.10,2.10]	1.62 [1.20,2.10]	1.80 [1.36,2.30]
Hemoglobin, g/dl	546 (14.6%)/684 (18.3%)	13.0 [11.8,14.1]	13.2 [11.9,14.4]	11.8 [10.7,13.0]	12.1 [10.9,13.3]
White blood cell count, 10^9^/L	52 (1.39%)/393 (10.5%)	8.96 [6.39,12.8]	10.0 [7.07,13.9]	8.30 [6.30,10.9]	9.82 [7.20,13.5]
Lymphocyte count, 10^9^/L	146 (3.90%)/407 (10.9%)	0.64 [0.42,0.91]	0.62 [0.41,0.90]	0.70 [0.48,1.05]	0.60 [0.40,0.90]
Neutrophil count, 10^9^/L	265 (7.08%)/468 (12.5%)	7.6 [5.20,11.3]	8.62 [5.96,12.5]	6.8 [4.92,9.20]	8.41 [5.90,11.9]
Platelet count, 10^9^/L	58 (1.55%)/331 (8.84%)	236 [181,312]	230 [174,302]	277 [212,350]	242 [179,316]
D-dimers, mg/L	705 (18.8%)/1191 (31.8%)	1197 [600,3398]	1371 [698,4110]	2000 [940,5174]	2498 [1102,7323]
Ferritin, ng/mL	2123 (56.7%)/2456 (65.6%)	1109 [561,1995]	1248 [678,1976]	994 [526,1690]	1214 [722,1928]
IL6, pg/mL	2747 (73.4%)/3112 (83.1%)	119 [38.3,299]	116 [40.1,272]	60.8 [15.4,242]	140 [35.5,555]
CRP, mg/dL	332 (8.87%)/700 (18.7%)	126 [58.1,215]	155 [72.3,249]	42.0 [17.1,95.1]	77.4 [26.4,193]
Procalcitonin, μg/l	1137 (30.4%)/1830 (48.9%)	0.20 [0.09,0.50]	0.27 [0.13,0.80]	0.12 [0.07,0.32]	0.36 [0.13,1.20]
Lactate dehydrogenase, U/l	612 (16.2%)/1105 (29.2%)	490 [376,652]	542 [414,740]	379 [304,492]	434 [338,585]
Troponin T, ng/l	3047 (81.4%)/3244 (86.7%)	0.01 [0.01,0.04]	0.02 [0.01,0.07]	0.02 [0.01,0.07]	0.02 [0.01,0.0
NT-proBNP, pg/mL	3132 (83.1%)/3330 (89%)	314 [123,785]	642 [245,1763	225 [108,567]	676 [239,2156]
Prothrombin time, seg	1239 (33.1%)/1474 (39.4%)	13.0 [12.1,14.2]	13.3 [12.2,14.7]	12.7 [11.9,13.9]	13.0 [11.9,14.4]
Bilirubin, mg/dL	476 (12.7%)/822 (22.0%)	0.60 [0.40,0.90]	0.60 [0.40,0.90]	0.50 [0.31,0.80]	0.60 [0.38,1.10]
Albumin, g/dL	1751 (46.8%)/1825 (48.8%)	3.10 [2.70,3.40]	3.10 [2.70,3.40]	2.88 [2.59,3.10]	2.64 [2.37,3.00]
Serum creatinine, mg/dL	42 (1.12%)/326 (8.71%)	0.75 [0.62,0.94]	0.93 [0.72,1.26]	0.70 [0.56,0.90]	0.99 [0.71,1.5
Sodium, mmol/L	467 (12.5%)/633 (16.9%)	139 [136,141]	138 [135,141]	142 [139,145]	141 [139,145]
Potassium, mmol/L	486 (13%)/640 (17.1%)	4 [3.66,4.40]	4.10 [3.70,4.50]	4.06 [3.70,4.40]	4.30 [3.90,4.70]
Tidal volume/PBW (mL/kg)	1264 (33.8%)/1155 (30.9%)	7.09 [6.38,7.91]	7.03 [6.38,7.81]	7.19 [6.46,8.0	7.08 [6.39,7.83]
PEEP, cmH_2_O	537 (14.3%)/366 (9.78%)	12 [10, 14]	12 [10, 14]	12 [10, 13]	12 [10, 14]
FiO_2_, %	100 (2.67%)/219 (5.85%)	80 [60,100]	90 [65,100]	45 [40,55]	55 [45,70]
Peak inspiratory pressure, cmH_2_O	1869 (49.9%)/1921 (51.0%)	30 [27, 34]	31 [28, 35]	28 [25, 32]	32 [28, 36]
End-inspiratory plateau pressure, cmH_2_O	2158 (57.7%)/2322 (61.3%)	24 [21, 27]	25 [22, 28]	23 [20, 26]	25 [22, 28]
Driving pressure, cmH_2_O^a^	2172 (58%)/2334(60%)	12 [10, 15]	12.5 [10, 15]	12 [9, 14]	12 [10, 15]
Compliance, mL/cmH_2_O^b^	2284 (61%)/2422 (64.7%)	35.8 [28.2,46.0]	35.7 [29.1,47]	37.8 [30,50]	36.4 [28.7,46.4]
Ventilatory ratio^c^	1346 (36%)/1279 (34.2%)	1.59 [1.28,1.95]	1.75 [1.40,2.24]	1.66 [1.40,1.99]	2.05 [1.69,2.50]
Neuromuscular blocking agent use	425 (11.4%)/555 (14.8%)	430 (35%)	1683 (81%)	551 (40%)	1394 (77%)
Position	681 (18.2%)/769 (20.5%)				
Supine		778 (70%)	1135 (58%)	946 (75%)	1048 (61%)
Prone		319 (28%)	770 (40%)	282 (23%)	631 (37%)
Other		18(2%)	42(2%)	29 (2%)	38 (2%)

Continuous variables are expressed as median (IQR) and categorical variables as numbers (percentages)

*CRP* C-reactive protein; *FiO*_*2*_ fraction of inspired oxygen; *IL* Interleukin; *MV* mechanical ventilation; *NT-proBNP* N-terminal pro-brain natriuretic peptide; *PaCO*_*2*_ arterial partial pressure of carbon dioxide; *PaO*_*2*_ partial pressure of arterial oxygen; *PBW* predicted body weight; *SOFA* sequential organ failure assessment score

^a^Defined as plateau pressure—PEEP

^b^Defined as tidal volume/ (Plateau pressure − PEEP)

^c^Defined as (minute ventilation × PaCO_2_)/ (PBW × 100 × 37.5)

**Fig. 3:**
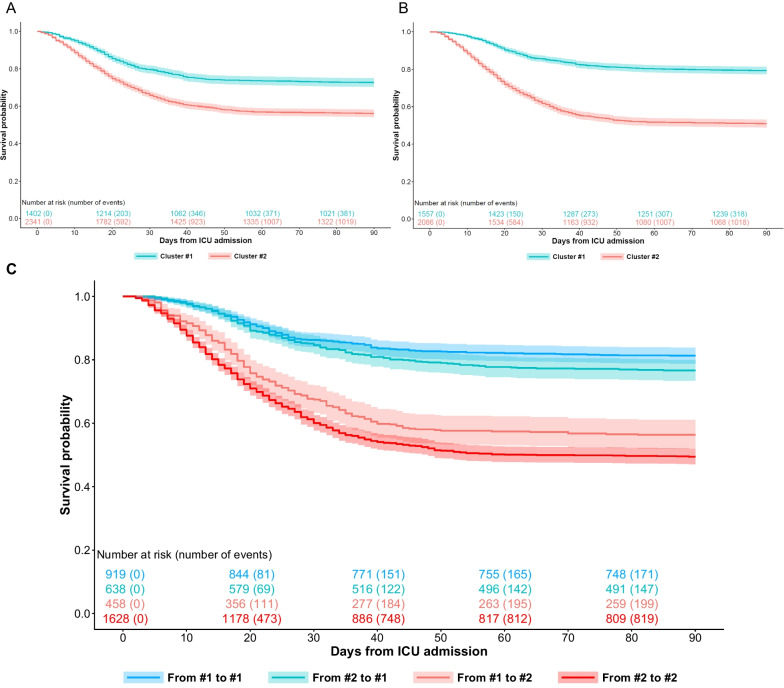
Unadjusted survival curves for clusters

The 90-day mortality for patients included in cluster 2 at baseline was 43.8% (*n* = 1025) versus 27.3% in cluster one (*n* = 383) (adjusted by SOFA score hazard ratio [aHR] 1.44, 95% confidence interval [95% CI] 1.21 to 1.70). The 90-day mortality was 49% (*n* = 1023) for patients included in cluster 2 on day 3 versus 20.6% (*n* = 321) for patients in cluster 1 (aHR 2.18, 95% CI 1.80–2.63). Unadjusted Kaplan–Meier survival curves are shown in Fig. 3.

**Fig. 4:**
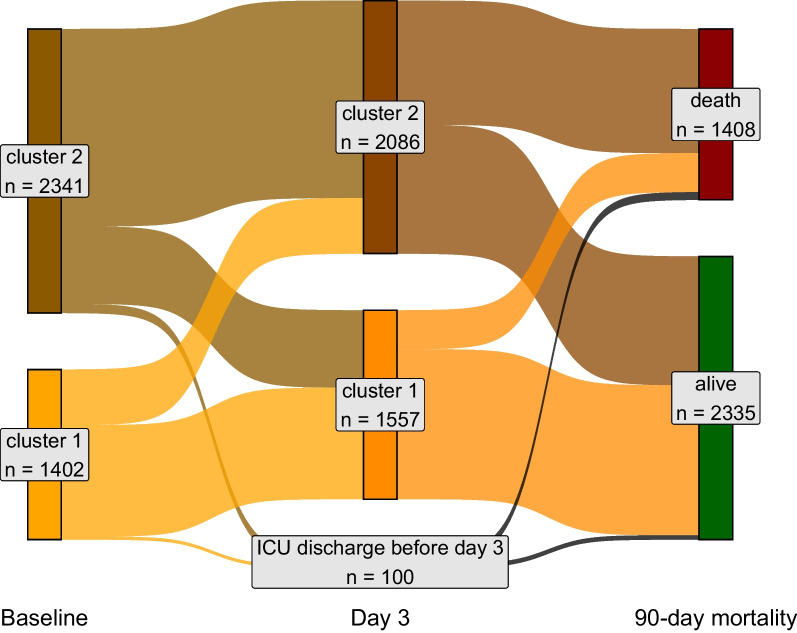
Sankey plot for ARDS population

Four hundred and fifty-eight (33%) patients clustered in the first group were clustered in the second group on day 3. In contrast, 638 (27%) patients clustered in the second group, were clustered in the first group on day 3. Lower survival probability at 90 days was observed in those patients grouped in cluster 2 at both time points compared to those who were grouped at cluster 1 at both time points (aHR 2.45, 95%CI 1.89–3.19), followed by those who changed from cluster 1 to cluster 2 over time (aHR 2.11, 95% CI 1.56–2.87). Those who changed from cluster 2 to cluster 1 overtime had similar survival probability (aHR 1.18, 95% CI 0.85–1.62) to those patients grouped at cluster 1 at both moments (Table 2, Figs. 3 and 4).

**Table 2:** Unadjusted hazard ratio (95% CI) for 90-day survival probability by clusters

	90-day mortality	Hazard ratio	95% CI
At baseline			
Cluster 1 (*n* = 1402)	383 (27.3%)	Reference	
Cluster 2 (*n* = 2341)	1025 (43.8%)	1.82	1.62–2.05
Day 3			
Cluster 1 (*n* = 1557)	321 (20.6%)	Reference	
Cluster 2 (*n* = 2086)	1023 (49.0%)	2.97	2.62–3.37
Change from baseline to day 3			
From 1 to 1 (*n* = 919)	172 (18.7%)	Reference	
From 1 to 2 (*n* = 458)	200 (43.7%)	2.77	2.26–3.40
From 2 to 1 (*n* = 638)	149 (23.4%)	1.26	1.02–1.58
From 2 to 2 (*n* = 1628)	823 (50.6%)	3.45	2.93–4.07

We conducted an evaluation to determine if we could cluster patients using parsimonious variables. To do this, we assessed the ability to group patients using C-index for SOFA score at baseline and on the third day. The results showed that the C-index was 0.74 (95% CI 0.72–0.76) for baseline and 0.79 (95% CI 0.77–0.81) for the third day. We further analyzed the results by selecting the four most differential variables of each clustering process (age, glucose, sodium bicarbonate and pH at baseline and ventilator ratio, peak inspiratory pressure, PaO_2_/FiO_2_, and pH on day 3). The C-index was 0.74 (95% CI 0.72–0.76) for baseline and 0.82 (95% CI 0.80–0.85) for the third day.

## Discussion

In our study, we found that patients with COVID-19-related ARDS could be clustered into two groups by k-prototypes at baseline and on day 3. Patients with more severe organ dysfunction, older age, worse oxygenation, respiratory mechanics, higher ventilatory ratio, pH, bicarbonate, and inflammatory markers were clustered in cluster 2 and had the highest mortality at both times. It is noteworthy that patients may change the cluster between the analyses at baseline or during day 3, improving or worsening their prognosis.

Strategies based on personalized medicine involve the identification of groups or clusters of patients with similar clinical and biological characteristics in order to tailor treatment to populations where it is most likely to be beneficial [28, 29]. This is usually not easy, as some conditions common to critically ill patients, such as sepsis or ARDS, are heterogeneous, share common causes and inflammatory pathways, and may differ in other ways. Clustering based on common clinical and biological characteristics has been proposed as a strategy to identify homogeneous populations to which treatments can be applied [30–32]. Post hoc analysis of negative clinical trials has shown that the same treatments may be beneficial in certain clusters. The clustering of ARDS patients was developed by cross-sectional analysis in the first hours of development; however, clinical characteristics and biological responses are dynamic and may change. In sepsis, a hyperinflammatory phase followed by a regulatory hypoinflammatory response has been described [33]. In pneumonia, markers of hyperinflammation may be followed by signs of immunosuppression, such as lymphocytopenia, with a more dysregulated response for both phases being associated with the worst prognosis [34, 35].

Typically, clinical trials aim to enroll patients within the first 24–48 h of admission. However, there may be a delay before treatment is administered during which the sub-phenotype classification of patients may change. In real-life situations, this delay may be even longer. Therefore, it is critical to continuously evaluate readily available measures that can dynamically classify patients and account for changes in sub-phenotypes. This is necessary because it affects prognosis. A previous study showed that sub-phenotypes analyzed by latent class analysis showed stability from baseline to day 3 and 94% remained in the same class [36]. Bos et al. did discover two trajectories related to ventilatory ratio and mechanical power that could potentially predict the duration of mechanical ventilation and the risk of death [16]. More recently, Chen and colleagues [37] described 3 longitudinal phenotypes with several changes between phenotypes during the first 4 days. Lu et al. analyzed the incidence of sub-phenotypes and their trajectories during 10 days according to respiratory support [38]. Sinha et al*.* observed that transcriptional profiling of the phenotypes reveals divergent biological signatures and changes in gene expression over time [39]. These results are in line with our findings and emphasize the need to classify patients just before planning an intervention taking into account the changes in clinical and analytical variables. According to our results, the probability of survival at 90 days is better predicted by clustering at day 3 (Fig. 3b, c), and the differences between clusters are also greater at day 3, but as expected with lower values compared to baseline (Table 1). Whether prognostic enrichment could be modified over time is also an important point to resolve.

Previous clustering processes were based on the inflammatory status, a fundamental key in the pathogenesis of ARDS. However, it is not well known how much inflammation is enough to damage the lungs. Whether or not a cytokine storm is associated with critical COVID-19 has been discussed in relation to anti-inflammatory treatment. It is recognized that in critically COVID-19 there is less inflammation than other diseases [40], although the impact on organ failure is large. It seems that the clustering process in our study is mostly influenced by organ failure, as suggested by the individual SOFA score components (as shown in Fig. 2). This is significant because the SOFA score is commonly used and can accurately predict how the subjects are assigned to clusters in our cohort. Changes in SOFA scores have been used to measure the outcome of several studies and as an indicator of poor prognosis. However, the prognosis of clustering in our cohort is independent of the SOFA score, as demonstrated by the adjusted HR.

We decided to use k-prototypes, an unsupervised machine-learning method for clustering. This method has shown good performance in identifying clusters for heterogeneous data [41] and allowed us to include continuous and categorical variables that we considered clinically relevant. Our results are similar to the clusters developed by latent class analysis in the order of inflammation, severity, respiratory parameters, and prognosis, and we found a significant difference in mortality between clusters at both time points evaluated.

It is important to acknowledge both the strengths and limitations of our study. On the positive side, we were able to collect data from a large and well-defined group of patients. However, it is important to note that our study focused only on patients with COVID-19-associated ARDS, and some results may be specific to COVID-19 rather than ARDS. However, most ARDS studies show pneumonia as the primary cause, which may help to mitigate this limitation. Other limitations are that we did not collect a complete biological, physiological or radiological assessment with cytokines, esophageal pressure or tomographic measurements for all patients. However, we were able to include several standard analytical variables and found clusters with similar characteristics as previously described. Furthermore, we included several variables that could vary according to the management of each center and also used all data as a derivation cohort and did not validate our results in another cohort. Finally, we found that the clustering process was different at baseline and day 3, with several variables changing their weight and the differences between clusters becoming more pronounced at day 3.

## Conclusion

During the first few days, patients can be clustered into two groups and the process of clustering patients may change as they continue to evolve. This means that despite a vast majority of patients remaining in the same cluster a minority reaching 33% of patients analyzed may be re-categorized into different clusters based on their progress. Such changes can significantly impact their prognosis. Such changes can significantly impact their prognosis, either positively or negatively. To ensure better classification, the clustering process must take into account the evolving condition of patients.

### Supplementary Information

**Additional file 1.** Clustering COVID-19 ARDS patients through the first days of ICU admission. An analysis of the CIBERESUCICOVID Cohort.

## Data Availability

The datasets used and/or analyzed during the current study are available from the corresponding author on reasonable request.

## Abbreviations

ARDS: Acute respiratory distress syndrome
HR: Hazard ratio
ICUs: Intensive care units
MV: Mechanical ventilation
PEEP: Positive end-expiratory pressure
SOFA: Sequential Organ Assessment Failure Score
