# Supplementary material for: Clustering COVID-19 ARDS patients through the first days of ICU admission. An analysis of the CIBERESUCICOVID Cohort
Source: Crit Care. 2024 Mar 21;28:91. doi: 10.1186/s13054-024-04876-5 (PMC10958830; doi:10.1186/s13054-024-04876-5)
Supplement: Supplementary file 1 — Additional file 1. Clustering COVID-19 ARDS patients through the first days of ICU admission. An analysis of the CIBERESUCICOVID Cohort. [file 13054_2024_4876_MOESM1_ESM.docx]

**CLUSTERING ARDS PATIENTS THROUGH THE FIRST DAYS OF ICU ADMISSION.**

**Supplementary.**

eFig 1. Correlation map.


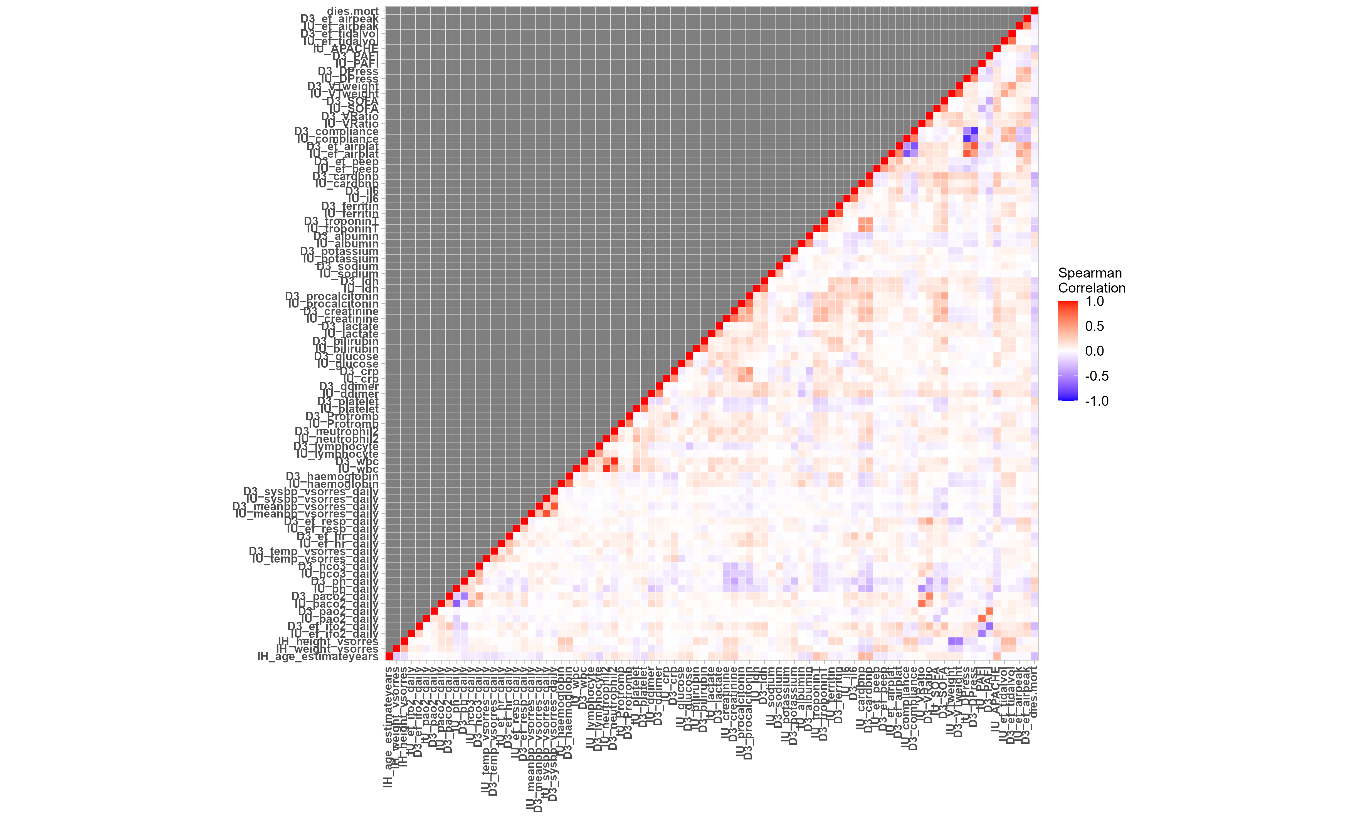


eFig2 Optics plot at baseline and day 3.


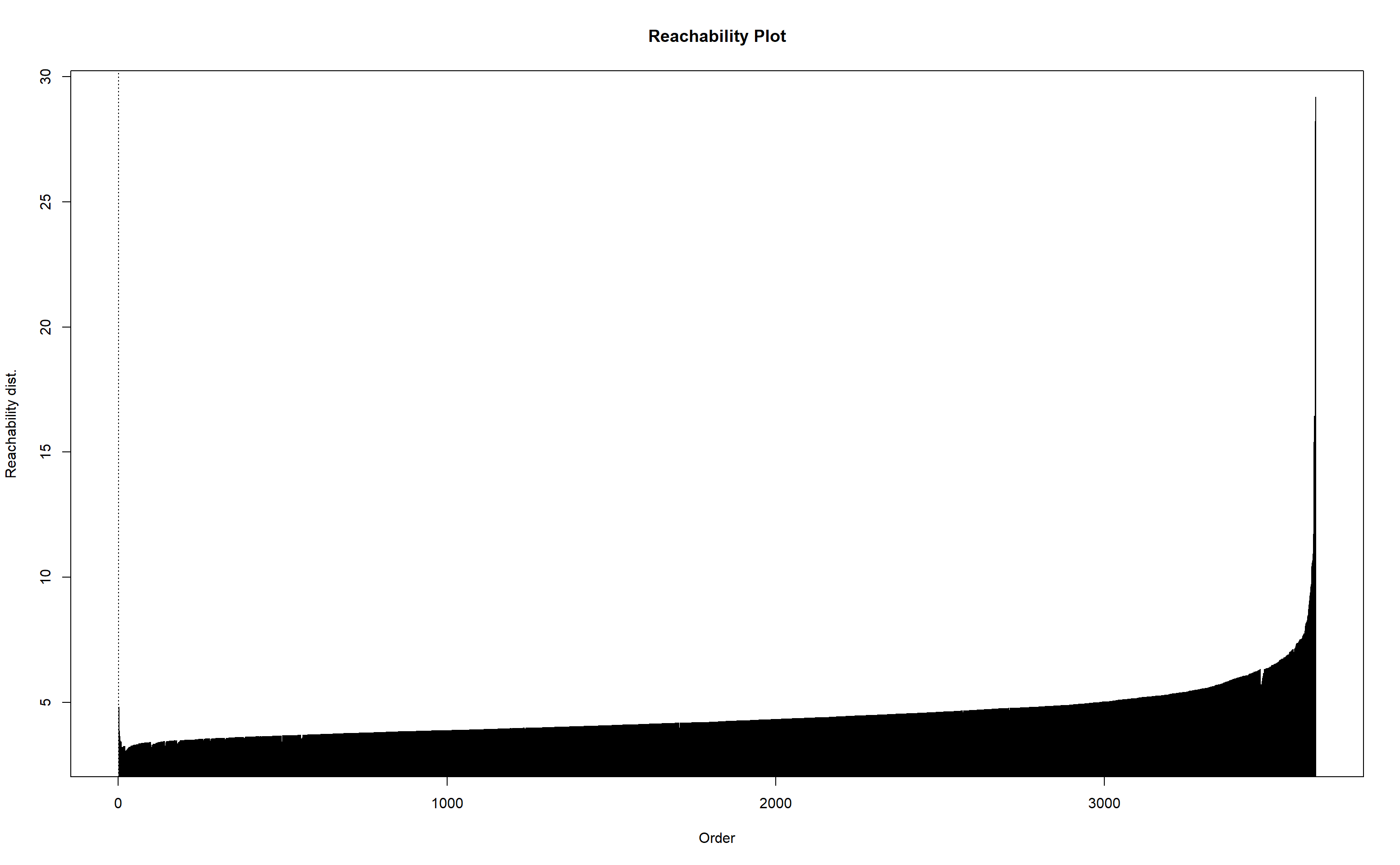

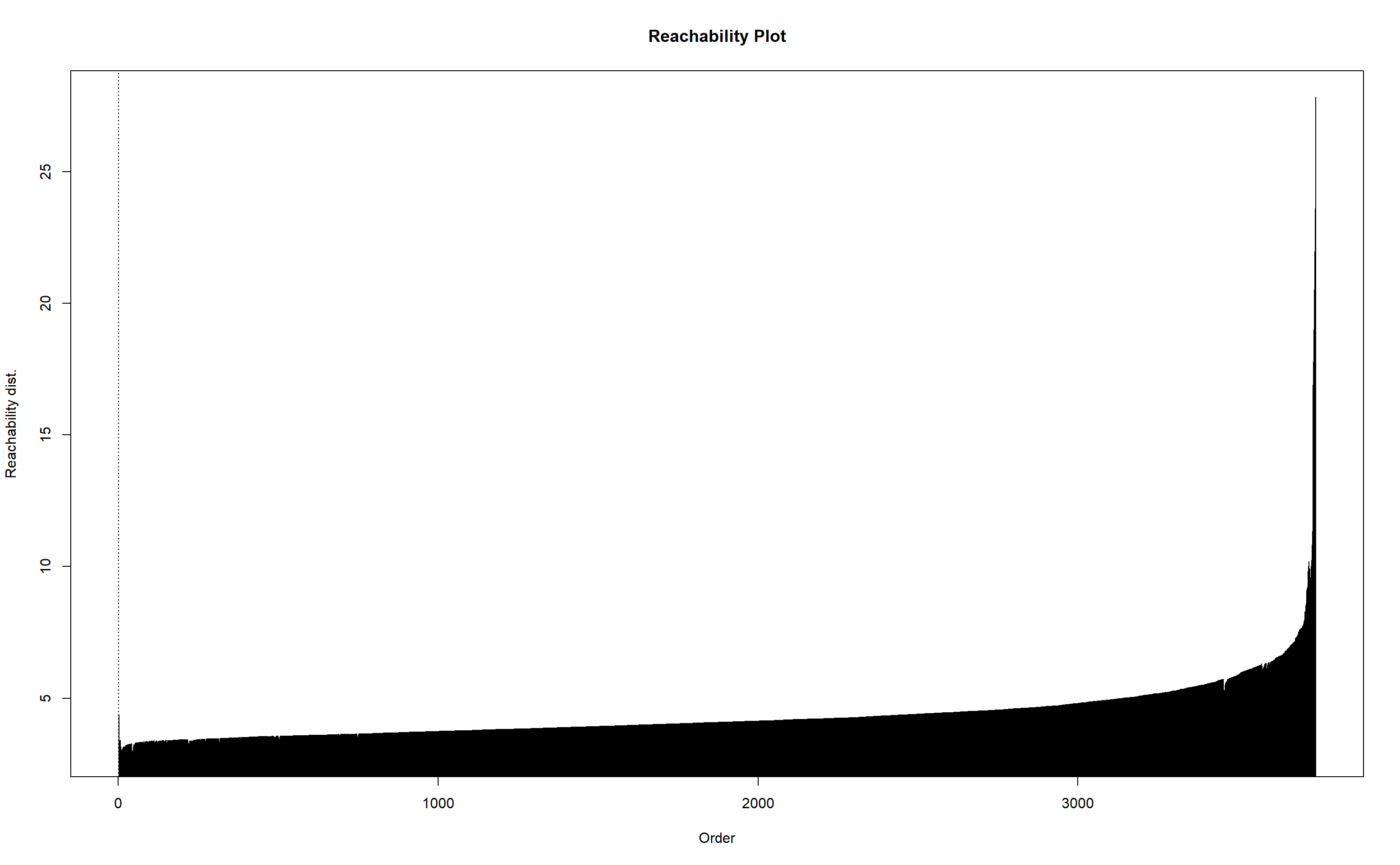


eFig3 Silhouette analysis at baseline and day 3.


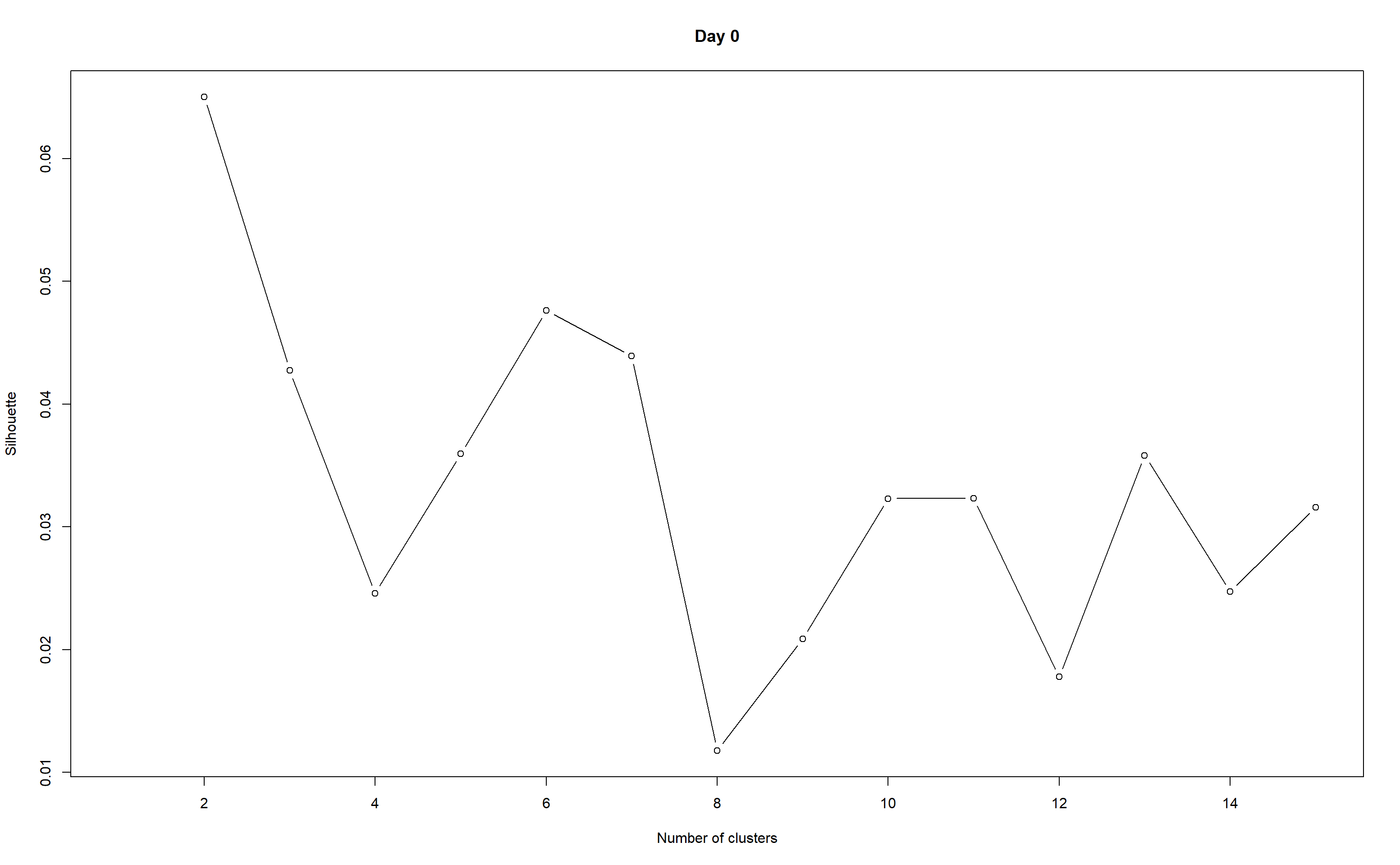

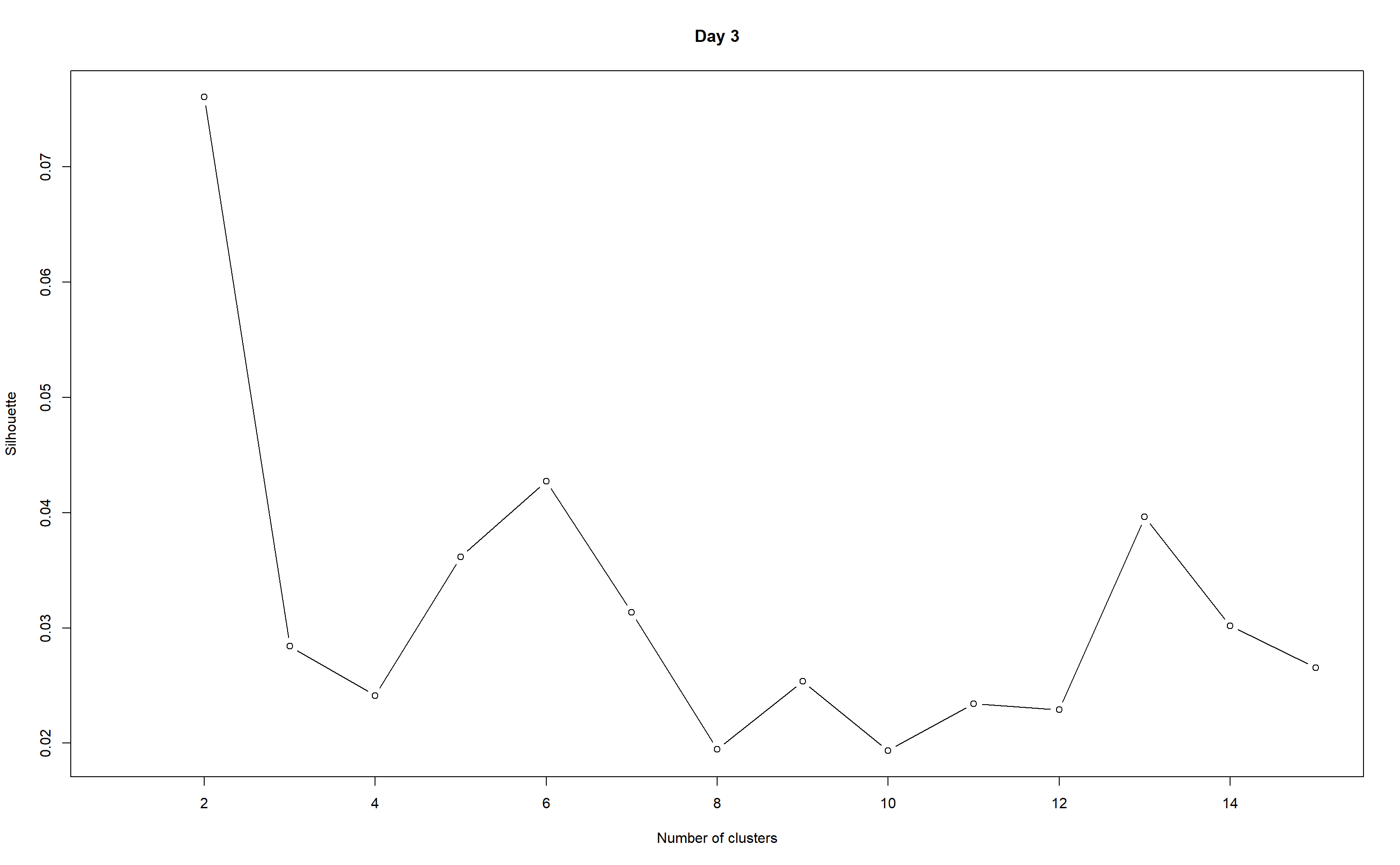


eTable 1 Cluster prototypes at baseline

IH_age_estimateyears IH_weight_vsorres IH_height_vsorres IU_ph_daily

1 -0.3793755 -0.10693159 -0.05921928 0.3310356

2 0.2272039 0.06404019 0.03546579 -0.1982537

IU_hco3_daily IU_temp_vsorres_daily IU_ef_hr_daily IU_ef_resp_daily

1 0.15830532 0.08539057 -0.03371615 0.03033819

2 -0.09480737 -0.05113950 0.02019224 -0.01816922

IU_meanbp_vsorres_daily IU_haemoglobin IU_wbc IU_lymphocyte IU_Protromb

1 0.09081503 -0.08113119 -0.11421128 -0.01828361 0.006163174

2 -0.05438816 0.04858861 0.06839992 0.01094986 -0.003691060

IU_platelet IU_ddimer IU_crp IU_glucose IU_bilirubin IU_lactate

1 0.04623228 0.05457389 -0.12520781 -0.2708070 0.03874958 -0.09644396

2 -0.02768802 -0.03268372 0.07498562 0.1621834 -0.02320671 0.05775926

IU_creatinine IU_procalcitonin IU_ldh IU_sodium IU_potassium

1 -0.2261653 -0.04320941 -0.14030546 0.08167753 -0.12262268

2 0.1354480 0.02587766 0.08402745 -0.04891580 0.07343742

IU_albumin IU_ef_peep IU_VRatio IU_VTweight IU_DPress IU_PAFI

1 -0.02210916 -0.1879883 -0.2039431 0.03412031 -0.03190679 0.13802012

2 0.01324094 0.1125842 0.1221393 -0.02043429 0.01910864 -0.08265878

IU_ef_airpeak IH_sex IH_chroniccard_mhyn IH_hypertension_mhyn IH_obesity_mhyn

1 -0.12699764 0 0 0 0

2 0.07605754 0 0 1 0

IH_diabetes_mhyn_2 IH_ia_haemaco IH_malignantneo_mhyn IH_ia_immune_other

1 0 0 0 0

2 0 0 0 0

IU_inotrop_cmtrt_daily IU_ef_position_daily IU_neuroblock_daily

1 0 0 0

2 1 0 1

IU_extracorp_prdur_daily

1 0

2 0

eTable 2 Cluster prototypes at day 3.

IH_age_estimateyears IH_weight_vsorres IH_height_vsorres D3_ph_daily

1 -0.3213753 -0.12291384 -0.1056538 0.4855043

2 0.2374982 0.09902101 0.0848044 -0.3623826

D3_hco3_daily D3_temp_vsorres_daily D3_ef_hr_daily D3_ef_resp_daily

1 0.12831748 -0.06082767 -0.1754510 -0.2084972

2 -0.09577676 0.04540205 0.1309574 0.1556233

D3_meanbp_vsorres_daily D3_haemoglobin D3_wbc D3_lymphocyte D3_Protromb

1 0.1779931 -0.08022788 -0.2191463 0.03184642 -0.07183932

2 -0.1328548 0.05988246 0.1635718 -0.02377031 0.05362120

D3_platelet D3_ddimer D3_crp D3_glucose D3_bilirubin D3_lactate

1 0.2006100 -0.09460849 -0.2806023 -0.2001558 -0.1545964 -0.12622519

2 -0.1497362 0.07061621 0.2094429 0.1493972 0.1153915 0.09421506

D3_creatinine D3_procalcitonin D3_ldh D3_sodium D3_potassium D3_albumin

1 -0.3486280 -0.12214536 -0.1967344 0.04782977 -0.2285391 0.1822899

2 0.2602176 0.09116986 0.1468435 -0.03570036 0.1705827 -0.1360620

D3_ef_peep D3_VRatio D3_VTweight D3_DPress D3_PAFI D3_ef_airpeak IH_sex

1 -0.2842000 -0.3538787 0.09197434 -0.06036396 0.2745598 -0.3518645 0

2 0.2121282 0.2641367 -0.06865007 0.04505594 -0.2049327 0.2626333 0

IH_chroniccard_mhyn IH_hypertension_mhyn IH_obesity_mhyn IH_diabetes_mhyn_2

1 0 0 0 0

2 0 1 0 0

IH_ia_haemaco IH_malignantneo_mhyn IH_ia_immune_other D3_inotrop_cmtrt_daily

1 0 0 0 0

2 0 0 0 1

D3_ef_position_daily D3_neuroblock_daily D3_extracorp_prdur_daily

1 0 0 0

2 0 1 0

| eTable 3Cox model of 90-day mortality by clusters and SOFA at baseline | | |
| --- | --- | --- |
|  | **Hazard ratio** | **95% CI** |
| cluster 2 (vs 1) | 1.4359 | (1.2127; 1.7002) |
| SOFA at day 0 | 1.1245 | (1.0865; 1.1637) |

**C-index (95% CI) = 0.602 (0.583 - 0.622)**

| eTable 4. Cox model of 90-day mortality by clusters and SOFA at day 3 | | |
| --- | --- | --- |
|  | **Hazard ratio** | **95% CI** |
| cluster 2 (vs 1) | 2.1773 | (1.8004; 2.633) |
| SOFA at day 3 | 1.1565 | (1.1198; 1.1945) |

**C-index (95% CI) = 0.679 (0.660 - 0.699)**

| eTable 5. Cox model of 90-day mortality by evolution of clusters from day 0 to day 3, SOFA at day 0 and change of SOFA from day 0 to day 3 | | |
| --- | --- | --- |
|  | **Hazard ratio** | **95% CI** |
| from 1 to 2 (vs from 1 to 1) | 2.1147 | (1.5562; 2.8737) |
| from 2 to 1 (vs from 1 to 1) | 1.1757 | (0.8506; 1.625) |
| from 2 to 2 (vs from 1 to 1) | 2.4539 | (1.886; 3.1928) |
| SOFA at day 0 | 1.1538 | (1.1059; 1.2037) |
| SOFA change | 1.1453 | (1.104; 1.1882) |

**C-index (95% CI) = 0.680 (0.659 - 0.700)**
